# Pierre Coulanges (1935-2024)

**DOI:** 10.48327/mtsi.v4i3.2024.565

**Published:** 2024-09-09

**Authors:** Jean-Philippe CHIPPAUX

**Affiliations:** SFMTSI Société francophone de médecine tropicale et santé internationale (ancienne SPE), Hôpital Pitié-Salpêtrière, Pavillon Laveran, 47-83 Boulevard de l'Hôpital, 75651 Paris cedex 13, France

Pierre Coulanges est décédé à son domicile de Fontaine Basse, à Gordes dans le département du Vaucluse le 17 mai 2024, à l’âge de 88 ans. Né le 14 août 1935 à Bollène (Vaucluse) dans une famille de médecins, Pierre Coulanges y passa ses jeunes années avant d'effectuer sa scolarité successivement à Toulon, Marseille et Bordeaux. De constitution fragile, il fut fréquemment malade au cours de cette période, manquant souvent l’école. Néanmoins, il passa son bac à 17 ans, puis le PCB1Le certificat d’études physiques, chimiques et naturelles (PCN), préparé dans les facultés des sciences, était indispensable pour entreprendre des études de médecine et pharmacie jusqu'en 1960. Il prépara à l’École annexe de Toulon le concours d'entrée à Santé navale de Bordeaux qu'il intégra en 1954. Il fut reçu au concours d'externat en 1958, puis compléta sa formation à l’École d'application du Service de santé des troupes coloniales du Pharo à Marseille.

**Figure 1:**
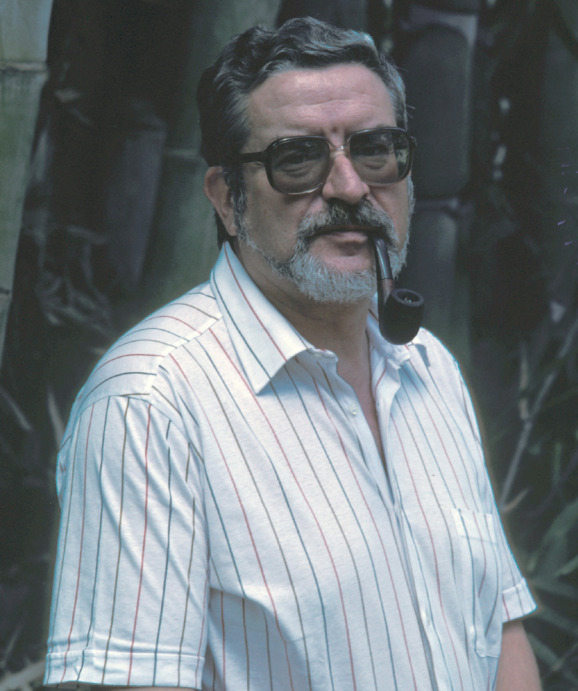
Pr. Pierre Coulanges, directeur de l'Institut Pasteur de Madagascar de 1975 à 1990 (crédit photo : Institut Pasteur)

En I960, il fut affecté comme médecin chef des arrondissements de Nioro du Sahel et Yélimané au Mali, un territoire de quelque 20 000 km2. Il arriva à Dakar en août 1960 et à Bamako en septembre, au moment de la tentative de coup d’État de Modibo Keita, de l’éclatement de la Fédération du Mali et de la proclamation de l'indépendance du Mali. Rompant les accords de coopération militaire avec la France, le nouveau chef d’État malien aligna progressivement le pays sur le modèle soviétique. Pour autant, Pierre Coulanges était « hors cadre des armées » – donc en emploi civil – et il put rejoindre son poste à Nioro du Sahel sans être inquiété. Il y pratiqua la médecine et la chirurgie tropicales – mais aussi traumatologiques et obstétricales – avec un succès certain qui lui valut la considération des autorités et députés locaux et le préserva des aléas politiques du moment… En août 1962, une brutale déshydratation, avec une perte de 13 kg en 24 heures, entraînant un coma et une insuffisance rénale aiguë, nécessita son évacuation sanitaire à l'hôpital principal de Dakar. Rétabli, il passa quelques mois au Sénégal comme médecin-chef du dispensaire civil de l'armée de terre, puis rejoignit Paris en mars 1963.

**Figure 2:**
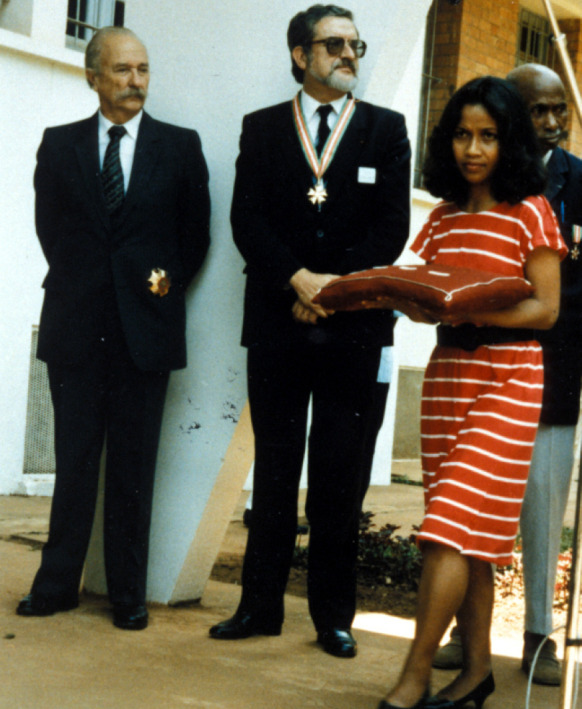
Manifestation à l'Institut Pasteur de Madagascar. Cérémonie pour le centenaire de la fondation de l'Institut Pasteur à Paris, le 29 octobre 1987. De gauche à droite : Pr. Edouard Brygoo, ancien directeur; Pr. Coulanges, directeur; et M. Edouard, préparateur (crédit photo : Institut Pasteur)

Il demanda à partir comme médecin-chef de l'archipel des Kerguelen où il résida de novembre 1963 à février 1965. Il y prépara le concours de l'assistanat des hôpitaux militaires qu'il passa en 1967. Il suivit le « grand cours » de l'Institut Pasteur en 1967-1968. En septembre 1968, il prit la direction du service de virologie de l'Institut Pasteur de Madagascar.

À son retour de Tananarive, en 1970, il fut nommé au laboratoire des arbovirus de l'Institut Pasteur de Dakar où il resta peu de temps. Rapatrié sanitaire à cause d'une tuberculose rénale, il fut hospitalisé six mois à l'hôpital d'instruction des armées Robert Piqué de Bordeaux, puis mis en congé de longue durée pendant six autres mois toujours à Bordeaux. Ce fut pour lui l'occasion de s'inscrire à la faculté de Bordeaux au certificat d’études supérieures (CES) d'anatomie pathologique qu'il obtint en 1973. Il suivit également le cours de mycologie médicale de l'Institut Pasteur en 1971. En 1972 et jusqu’à mi-1973, il fut médecin-chef du laboratoire de l'hôpital maritime de Rochefort-sur-mer.

Pierre Coulanges retourna à l'Institut Pasteur de Madagascar en octobre 1973 comme chef du laboratoire de virologie avant d’être nommé sousdirecteur le 22 novembre 1973 puis directeur le 1^er^ mai 1975. Alors qu'il était en mission à Dakar, sa femme décède d'une septicémie à l'hôpital Girard et Robic de Tananarive. Malgré les difficultés politiques et économiques que connaissait Madagascar à cette époque, Pierre Coulanges a fortement contribué à la modernisation et au développement de cet Institut. En désaccord avec la direction de l'Institut Pasteur à Paris, dont celui de Madagascar était une filiale, désavoué par le Service de santé des armées, il prit sa retraite anticipée au grade de médecin-chef des services le 31 décembre 1990.

Resté quelques temps à Madagascar, il s'installa en 1992 à Gordes où il s'est éteint.

Forte personnalité au caractère bien trempé, il réussit à assumer les missions dont il avait la charge sans rien céder aux contingences politiques tant au Mali qu’à Madagascar. Il faisait preuve d'une double loyauté, essentielle pour tout coopérant, à son pays tout autant qu’à celui pour lequel il était mis à disposition. Cette qualité lui assurait le respect et la confiance du personnel local et expatrié et des autorités des deux pays. Excellent administrateur, animé d'une curiosité scientifique appliquée aussi bien au terrain qu'au laboratoire, ces qualités lui ont permis de développer l'Institut Pasteur de Madagascar dans des conditions extrêmement difficiles. Homme de culture, il était fidèle en amitié et profondément humain envers les plus vulnérables.

Spécialiste dans de nombreuses disciplines – anatomo-pathologiste, biologiste des hôpitaux des armées, microbiologiste, immunologiste –, il est auteur ou co-auteur de quelque 200 articles, dont 24 dans le *Bulletin de la Société de pathologie exotique,* communications et rapports d'enquête ou de recherche. Sa contribution scientifique lui valut la médaille de bronze du Service de santé des armées pour travaux scientifiques et techniques. Pierre Coulanges était membre de l'Académie malgache, membre correspondant de l'Académie des sciences d'outre-mer, membre de la Société de pathologie exotique, de la Société internationale de standardisation biologique, de la Société française de microbiologie, de la Société française de mycologie humaine, de l'Association des épidémiologistes de langue française, de la Société des sciences médicales de Madagascar – dont il a été vice-président – et de l'Association des anciens élèves de l'Institut Pasteur.

Il était chevalier de la Légion d'honneur, officier de l'Ordre national du Mérite et grand-croix de l'Ordre national malgache.
