# Supplementary material for: Pierre Coulanges (1935-2024)
Source: Med Trop Sante Int. 2024 Sep 9;4(3):mtsi.v4i3.2024.565. [Article in French] doi: 10.48327/mtsi.v4i3.2024.565 (PMC11809064; doi:10.48327/mtsi.v4i3.2024.565)
Supplement: Related texts

# Research article: Pierre Coulanges (1935-2024)

Pierre Coulanges died at his home in Fontaine Basse, Gordes, Vaucluse, on May 17, 2024, at the age of 88. Born on August 14, 1935 in Bollène (Vaucluse) into a family of doctors, Pierre Coulanges spent his early years there before attending school in Toulon, Marseille and Bordeaux. Weakly built, he was frequently ill during this period, often missing school. Nevertheless, he passed his baccalaureate at the age of 17, then the PCB1The certificate of physical, chemical and natural studies (PCN), prepared in the faculties of science, was essential for studying medicine and pharmacy until 1960. He prepared for the competitive entrance examination for the Santé navale de Bordeaux at the École annexe de Toulon, which he joined in 1954. He passed the externat examination in 1958, then completed his training at the École d'application du Service de santé des troupes coloniales du Pharo in Marseille.

**Figure 1:** Prof. Pierre Coulanges, Director of the Institut Pasteur de Madagascar from 1975 to 1990 (photo credit: Institut Pasteur)

In 1960, he was appointed chief medical officer for the districts of Nioro du Sahel and Yélimané in Mali, a territory covering some 20,000 km2. He arrived in Dakar in August 1960 and in Bamako in September, at the time of Modibo Keita's attempted coup d’état, the break-up of the Federation of Mali and the proclamation of Mali's independence. Breaking off military cooperation agreements with France, Mali's new head of state gradually aligned the country with the Soviet model. Nevertheless, Pierre Coulanges was “hors cadre des armées” - i.e., in civilian employment - and was able to return to his post in Nioro du Sahel without being troubled. There, he practiced tropical medicine and surgery - as well as traumatology and obstetrics - with great success, earning the esteem of local authorities and deputies, and shielding him from the political ups and downs of the time… In August 1962, a sudden bout of dehydration, with a loss of 13 kg in 24 hours, leading to a coma and acute renal failure, necessitated his medical evacuation to the main hospital in Dakar. After recovering, he spent a few months in Senegal as head physician of the army's civilian dispensary, before returning to Paris in March 1963.

**Figure 2:** Celebration at the Institut Pasteur in Madagascar. Centennial celebration of the Institut Pasteur in Paris, October 29, 1987. From left to right: Pr. Edouard Brygoo, former director; Pr. Coulanges, director; and M. Edouard, preparator (photo credit : Institut Pasteur)

He asked to go to the Kerguelen Islands as Chief Medical Officer, where he stayed from November 1963 to February 1965. There he prepared for the competitive examination for military hospital assistants, which he passed in 1967. He attended the “grand cours” of the Institut Pasteur in 1967-1968. In September 1968, he became head of the virology department at the Pasteur Institute in Madagascar.

On his return from Tananarive in 1970, he was appointed to the arbovirus laboratory at the Pasteur Institute in Dakar, where he remained for a short time. Repatriated for medical reasons due to renal tuberculosis, he was hospitalized for six months at the Robert Piqué Army Training Hospital in Bordeaux, then placed on long-term leave for another six months, again in Bordeaux. This gave him the opportunity to enroll in the Certificate of Advanced Studies (CES) in Pathological Anatomy at the Bordeaux Faculty of Medicine, which he obtained in 1973. He also attended the medical mycology course at the Institut Pasteur in 1971. In 1972 and until mid-1973, he was head of the laboratory at the Rochefort-sur-mer naval hospital. Pierre Coulanges returned to the Institut Pasteur de Madagascar in October 1973 as head of the virology laboratory, before being appointed deputy director on November 22, 1973, and then director on May 1, 1975. During a mission in Dakar, his wife died of sepsis at the Girard et Robic Hospital in Tananarive. Despite the political and economic difficulties facing Madagascar at the time, Pierre Coulanges made a major contribution to the modernization and development of the Institute. In disagreement with the management of the Institut Pasteur in Paris, of which the institute in Madagascar was a subsidiary, and disavowed by the Army Health Service, he took early retirement with the rank of Chief Medical Officer on December 31, 1990.

After spending some time in Madagascar, he settled in Gordes in 1992, where he died.

A strong personality with a strong character, he managed to carry out the missions for which he was responsible without succumbing to the political contingencies in Mali and Madagascar. He demonstrated the dual loyalty that is essential for any aid worker, both to his own country and to the country to which he was assigned. This earned him the respect and trust of local and expatriate staff, as well as the authorities in both countries. An excellent administrator, driven by scientific curiosity applied both in the field and in the laboratory, these qualities enabled him to develop the Institut Pasteur de Madagascar under extremely difficult conditions. A man of culture, he was loyal in friendship and deeply humane towards the weakest.

A specialist in many disciplines - anatomopathologist, army hospital biologist, microbiologist, immunologist - he authored or co-authored some 200 articles, including 24 in the *Bulletin de la Société de Pathologie Exotique,* as well as communications and investigation or research reports. His scientific contribution earned him the bronze medal of the French Armed Forces Health Service for scientific and technical work.

Pierre Coulanges was a member of the Académie Malgache, a corresponding member of the Académie des Sciences d'Outre-Mer, a member of the Société de Pathologie Exotique, the Société Internationale de Standardisation Biologique, the Société Française de Microbiologie, the Société Française de Mycologie Humaine, the Association des Epidémiologistes de Langue Française, the Société des Sciences Médicales de Madagascar - of which he was Vice-President - and the Association des Anciens Élèves de l'Institut Pasteur.

He was a chevalier of the Légion d'honneur, an officier of the Ordre national du Mérite, and a grand-croix of the Ordre national malgache.
